# Electrogenerated poly(pyrrole-lactosyl) and poly(pyrrole-3'-sialyllactosyl) interfaces: toward the impedimetric detection of lectins

**DOI:** 10.3389/fchem.2013.00010

**Published:** 2013-07-12

**Authors:** Chantal Gondran, Marie-Pierre Dubois, Sébastien Fort, Serge Cosnier

**Affiliations:** ^1^Département de Chimie Moléculaire (DCM-UMR CNRS 5250), Institut de Chimie Moléculaire de Grenoble, (ICMG-FR CNRS 2607), Université Joseph FourierGrenoble, France; ^2^Centre de Recherche sur les Macromolécules Végétales (CERMAV-UPR CNRS 5301), Institut de Chimie Moléculaire de Grenoble, (ICMG-FR CNRS 2607)Grenoble, France

**Keywords:** oligosaccharide, lectin, electropolymerization, polypyrrole, electrochemical impedance spectroscopy, biosensor

## Abstract

This paper reports on the impedimetric transduction of binding reaction between polymerized saccharides and target lectins. The controlled potential electro-oxidation of pyrrole-lactosyl and pyrrole-3′-sialyllactosyl at 0.95 V vs. Ag/AgCl, provides thin and reproducible poly(pyrrole-saccharide) films. The affinity binding of two lectins: *Arachis hypogaea*, (PNA) and *Maackia amurensis* (MAA) onto poly(pyrrole-lactosyl) and poly(pyrrole-3′-sialyllactosyl) electrodes, was demonstrated by cyclic voltammetry in presence of ruthenium hexamine and hydroquinone. In addition, rotating disk experiments were carried out to determine the permeability of both polypyrrole films and its evolution after incubating with lectin target. Finally, the possibility of using the poly(pyrrole-lactosyl) or poly(pyrrole-3′-siallyllactosyl) films for the impedimetric transduction of the lectin binding reaction, was investigated with hydroquinone (2 × 10^−3^ mol L^−1^) as a redox probe in phosphate buffer. The resulting impedance spectra were interpreted and modeled as an equivalent circuit indicating that charge transfer resistance (*R*_ct_) and relaxation frequency (f°) parameters are sensitive to the lectin binding. *R*_ct_ increases from 77 to 97 Ω cm^2^ for PNA binding and from 93 to 131 Ω cm^2^ for MAA binding. In parallel, f° decreases from 276 to 222 Hz for PNA binding and from 223 to 131 Hz for MAA binding. This evolution of both parameters reflects the steric hindrances generated by the immobilized lectins towards the permeation of the redox probe.

## Introduction

Lectins, non-immunoglobulin proteins that bind carbohydrates, play a central role in a wide range of biological processes such as cell–cell recognition, viral and bacterial pathogenesis, and inflammation (Sharon, 2007). In addition to continuous progresses in oligosaccharide synthesis, sensitive and label-free detection of carbohydrate-lectin interactions has emerged over the last decade as an important diagnostic tool in medical or food applications (Zeng et al., 2012). Among others, a biosensor functionalized with blood group P related disaccharide showed high specificity in the binding of the uropathogenic bacteria P-fimbriated *Escherichia coli* compared to the binding of non-infectious bacteria (Nilsson and Mandenius, 1994; Furtado et al., 2012). More recently, a xyloglucan film-based biosensor have been developed for the toxicity assessment of ricin in castor seed meal (Stevens et al., 2006).

Lectin detection has been investigated using different methods such as QCM (Shen et al., 2007; Wilczewski et al., 2008; Norberg et al., 2011, 2012), fluorescence (Kikkeri et al., 2009; Sanji et al., 2009; Shiraishi et al., 2010; Mandal et al., 2012), SPR (Vornholt et al., 2007; Gondran et al., 2008; Murthy et al., 2008; Wilczewski et al., 2008; Spain and Cameron, 2011), voltammetry (Casas-Solvas et al., 2008; Hu et al., 2012), electrochemical impedance spectroscopy (EIS) (Dubois et al., 2005; Szunerits et al., 2010; Xi et al., 2011; Oliveira et al., 2011). EIS is an efficient and sensitive technique suitable for the characterization of deposits on electrode surfaces and their evolution. This technique is especially promising for the development of affinity biosensors. Indeed, affinity biosensors are based on a molecular recognition process such as DNA hybridization, immunoreaction or ligand–protein interactions that could affect the properties of the electrode surface. Moreover, this approach allows label-free detection in bioanalysis since it bypasses the need to modify biomolecules with fluorescent or enzymatic labels (Daniels and Pourmand, 2007; Baur et al., 2010; Sanchez-Pomales and Zangmeister, 2011). However, EIS method requires a reversible signal from a redox probe diffusing from the solution to the electrode surface through the recognition layer. Consequently, this method necessitates the reproducible immobilization of a recognition layer on the surface of the electrode which has a high permeability to allow the diffusion of redox probe. Taking into account that lectins are much smaller proteins than antibodies, the recognition layer should display higher densities of carbohydrate-sensing elements than conventional configurations of immunosensors while maintaining a high permeability.

The stable and reproducible immobilization of saccharides on a surface with complete retention of their biological activity remains always a crucial problem. Different methods for engineering surfaces that contain an ordered arrangement of selected carbohydrates have been reported (Dyukova et al., 2006; Rillahan and Paulson, 2011). Among the various methods of electrode modification, the electrogeneration of functionalized polymers as recognition interfaces between an electrode and the lectin target is a promising avenue due to the reproducibility of the polymer formation and the easy control of its thickness and morphology (Cosnier, 2007; Mercey et al., 2008).

**Figure 1:**
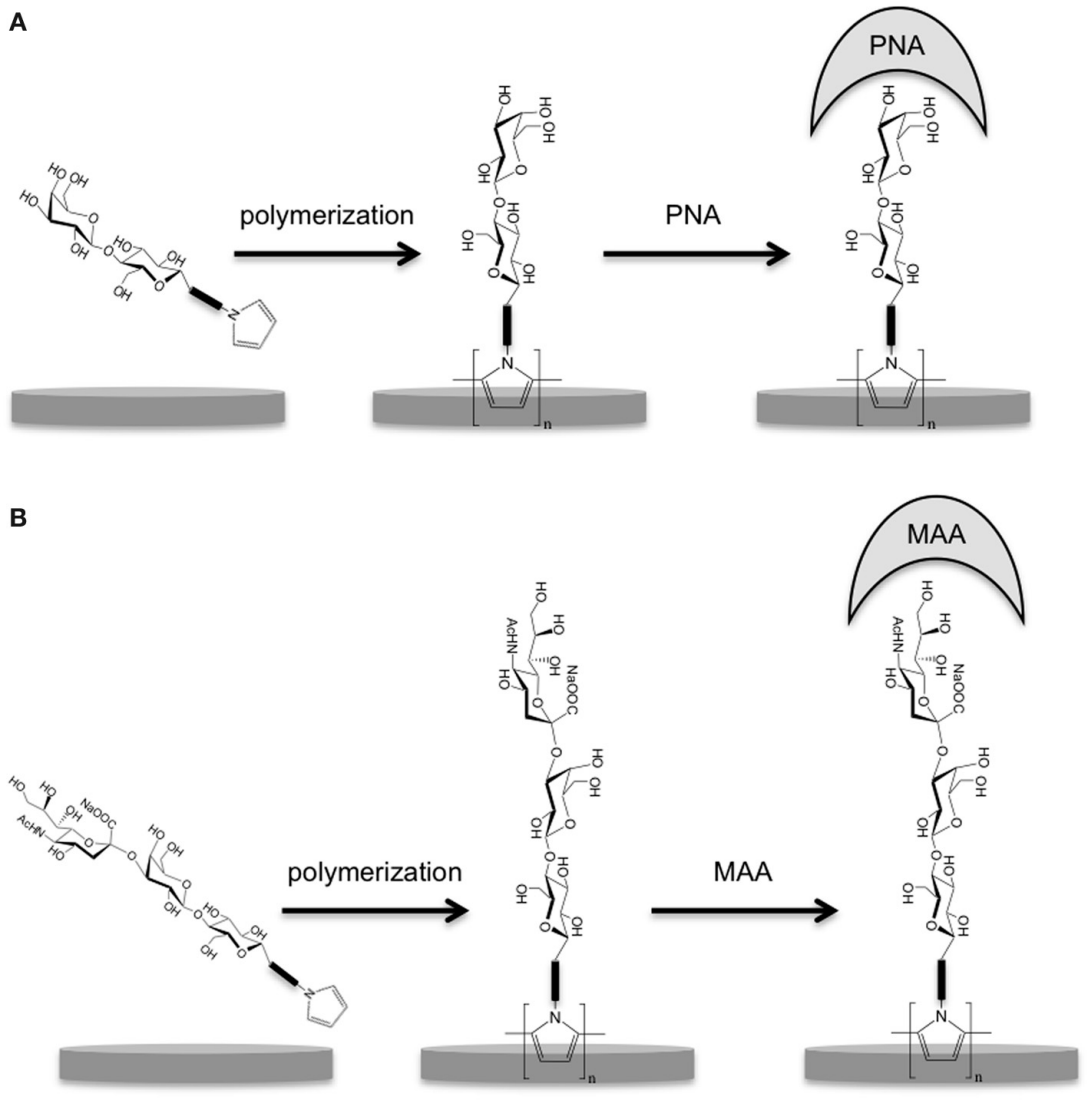
**Schematic representation of (A) poly(pyrrole-lactosyl) film formed on electrodes by electropolymerization of pyrrole-lactosyl and detection of PNA; (B) poly(pyrrole-3′-sialyllactosyl) film formed on electrodes by electropolymerization of pyrrole-lactosyl and detection of MAA**.

We, previously, reported the synthesis of pyrrole-functionalized oligosaccharides: pyrrole-lactosyl and pyrrole-3′-sialyllactosyl and their use for the generation of SPR sensors specific to two lectins (*Arachis hypogaea*, PNA and *Maackia amurensis* MAA agglutinins) (Gondran et al., 2008). In this context, we report here the electrochemical characterization of these carbohydrate-polymers and the investigations of their potentialities for the impedimetric detection of lectins and hence for the development of label-free impedance sensors (Figure 1). Indeed, rapid, sensitive and cost-effective detection of lectin binding specifically to α-2,3 or 2,6 sialylated oligosaccharides are highly desirable diagnostic tools for specific detection of influenza viruses. For instance, while human flu viruses prefer to bind to receptors that display sialic acid linked to galactose by an α-2,6 linkage, highly pathogenic avian flu viruses prefer to bind to the α-2,3 linkage (Stevens et al., 2006).

## Materials and methods

### Chemicals

Lithium perchlorate (LiClO_4_) was purchased from Fluka and was used as received. Hydroquinone was purchased from Prolabo (VWR international) and ruthenium hexamine trichloride from Aldrich. Pyrrole-lactosyl and pyrrole-3′-sialyllactosyl were synthesized as described in previous publications (Fort et al., 2005; Gondran et al., 2008). PNA and MAA agglutinins were obtained from Sigma. All other chemicals were analytical reagent grade and obtained from Aldrich. All solutions were prepared with Millipore water (18 MΩ cm).

### Electrochemical instrumentation

Electrochemical investigations were performed with Autolab potentiostat 100 (EcoChemie, Utrecht, The Netherlands) and a conventional three-electrode configuration was employed in all experiments. Glassy carbon disk electrodes (3 or 5 mm of diameter) polished with 1 μm diamond paste were used as working electrode. A platinum wire placed in a separated compartment containing the supporting electrolyte was used as an auxiliary electrode. A saturated Ag-AgCl-KCl electrode (Ag/AgCl) was used as reference electrode for voltammetry experiments while a saturated calomel electrode (SCE) was employed for impedance measurements.

As previously described (Gondran et al., 2008), polymerization of the sensing polymers were carried out by electrochemical oxidation of the pyrrole group of pyrrole-lactosyl or pyrrole-3′-sialyllactosyl into radical cation and their subsequent coupling. Potentiostatic chronocoulometries at +0.95 V were performed with pyrrole-lactosyl or pyrrole-3′-sialyllactosyl (3 × 10^−3^ mol L^−1^) in H_2_O + 0.1 mol L^−1^ LiClO_4_. Polymerization processes are stopped for a charge of 10^−4^ C. Cyclic voltammetry is then used to obtain the signal of the polymer formed in H_2_O + 0.1 mol L^−1^ LiClO_4_ solution free of monomer. Voltammograms were recorded at 0.1 V s^−1^.

Cyclic voltammetries were carried out with hydroquinone (2 × 10^−3^ mol L^−1^) or Ru(NH_3_)_6_^3+^ (2 × 10^−3^ mol L^−1^) as a redox probe in 0.1 mol L^−1^ phosphate buffer solution (pH 7.2). Voltammograms were recorded at 0.1 V s^−1^.

Permeability measurements were carried out with hydroquinone (2 × 10^−3^ mol L^−1^) as a redox probe in 0.1 mol L^−1^ phosphate buffer solution (pH 7.2). The rotating disk voltammograms were recorded at 5 × 10^−3^ V s^−1^, the rotating rate varying from 250 to 3000 rpm.

The electrochemical impedance measurements were performed at room temperature by immersing the electrode into 0.1 mol L^−1^ phosphate buffer (pH 7.2) containing hydroquinone (2 × 10^−3^ mol L^−1^) as a redox probe. The frequency sweep was from 50 kHz to 0.01 Hz. Impedance measurements were performed at a potential of 0.35 V/SCE with AC amplitude of 5 mVrms at a rotation rate of 3000 rpm. A ZView software (Scribner Associates Inc.) was used to simulate the data based on an appropriate equivalent electrical circuit.

### Lectin assays

The binding of lectin was studied by incubating of 20 μl of PNA or MAA (8.3 μmol L^−1^) in phosphate buffer (0.1 mol L^−1^, pH 7.2) containing 10^−3^ mol L^−1^ of CaCl_2_ and 10^−3^ mol L^−1^ of MnCl_2_, for 45 min at room temperature. Electrodes were then rinsed with phosphate buffer (0.1 mol L^−1^, pH 7.2).

## Results and discussion

### Electrochemical elaboration and characterization of poly(pyrrole-lactosyl) and poly(pyrrole-3′-sialyllactosyl) films on glassy carbon electrodes

**Figure 2:**
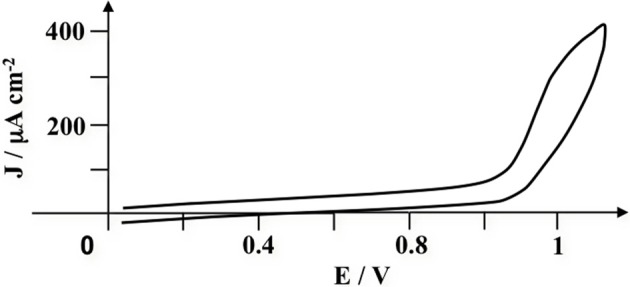
**Cyclic voltammogram recorded at a glassy carbon electrode of pyrrole-lactosyl (3 × 10^−3^ mol L^−1^) in H_2_O + 0.1 mol L^−1^ LiClO_4_.** Scan rate = 0.1 V s^−1^.

The electrochemical behavior of the pyrrole-lactosyl (3 × 10^−3^ mol L^−1^) was investigated in H_2_O + 0.1 mol L^−1^ LiClO_4_. Upon oxidative scanning, the monomer exhibits an irreversible peak at 1.0 V corresponding to the oxidation of the pyrrole group in highly reactive radical cation in order to form polypyrrole film (Figure 2). The potential of the electrochemical oxidation of pyrrole-lactosyl is in accordance with those previously reported for N-alkyl-pyrrole monomers (0.8–1.1 V) (Diaz et al., 1982). The cyclic voltammogram of pyrrole-3′-sialyllactosyl recorded under the same conditions shows a similar behavior with an identical potential value for the irreversible oxidation peak (data not shown).

**Figure 3:**
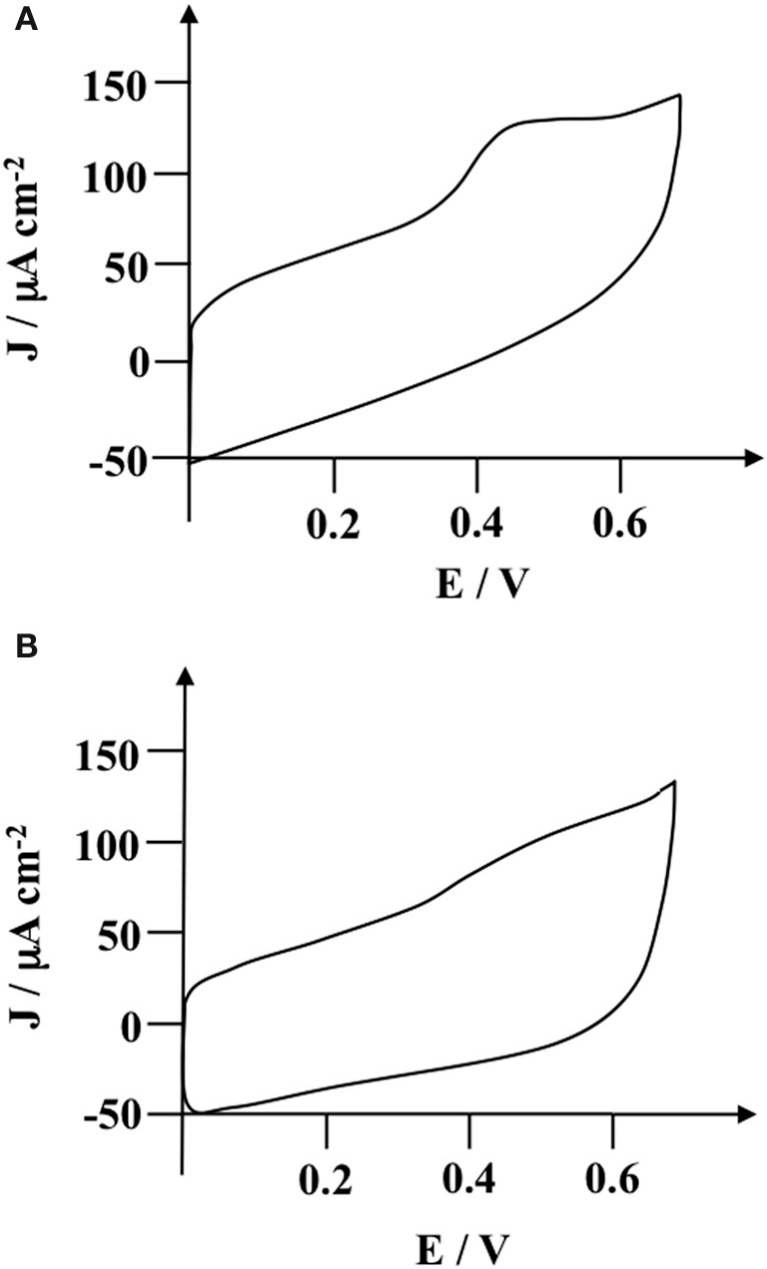
**(A)** Cyclic voltammogram of the poly(pyrrole-lactosyl) film deposited on a glassy carbon electrode (Γ = 3.2 × 10^−9^ mol cm^−2^) in H_2_O + 0.1 mol L^−1^ LiClO_4_; **(B)** cyclic voltammogram of the poly(pyrrole-3′-sialyllactosyl) film (Γ = 2.8 × 10^−9^ mol cm^−2^) in H_2_O + 0.1 mol L^−1^ LiClO_4_. Scan rate = 0.1 V s^−1^.

In order to generate thin and reproducible polymeric layers, electropolymerization of pyrrole-lactosyl and pyrrole-3′-sialyllactosyl was performed by controlled potential electrolysis at 0.95 V on glassy carbon electrode (passed charge: 10^−4^ C). The resulting electrodes were then transferred into H_2_O + 0.1 mol L^−1^ LiClO_4_ solution free of monomer. As expected the cyclic voltammograms of these electrodes exhibit a broad oxidation peak around 0.4 V assigned to the oxidation of the polypyrrolic matrix (Figure 3). It appears that the peak of polypyrrole oxidation is more clearly defined for poly(pyrrole-lactosyl) suggesting a better electroactivity of this film. In fact, the higher solubility and bulkiness of pyrrole-siallylactose compared to pyrrole-lactosyl may promote the formation of short oligomers instead of long polymeric chains and hence led to a lower polymer conductivity. The apparent surface coverages of the electropolymerized pyrrole-lactosyl and pyrrole-3′-sialyllactosyl (Γ = 3.2 × 10^−9^ and 2.8 × 10^−9^ mol cm^−2^), were determined from the charge recorded under the oxidation wave of the poly(pyrrole) backbone. Different polymerization charges were used to evaluate the efficiency of the polymerization process for both monomers. A similar behavior was observed, the electric yield decreasing from 75% to about 50% for respectively 0.5 × 10^−4^ and 1 × 10^−4^ C, reaching then 10% for an electrolysis charge of 5 × 10^−4^ C.

### Cyclic voltammetric characterization of poly(pyrrole-lactosyl)/PNA and poly(pyrrole-3′-sialyllactosyl)/MMA interfaces

**Figure 4:**
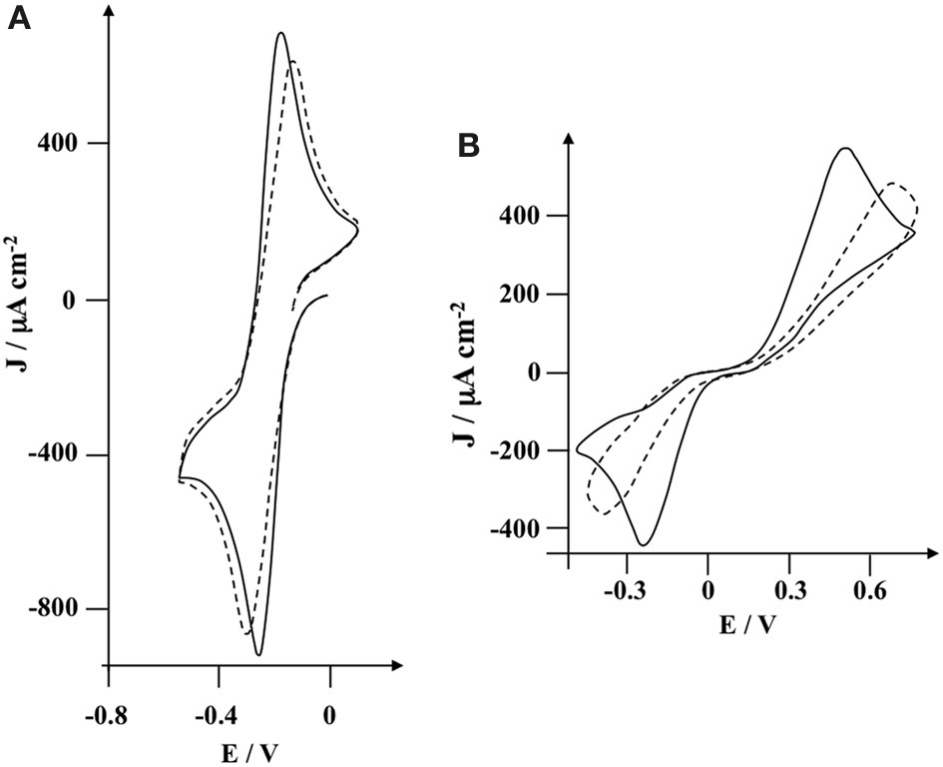
**Cyclic voltammogram of (A) ruthenium hexamine (2 × 10^−3^ mol L^−1^) (B) hydroquinone (2 × 10^−3^ mol L^−1^) at a glassy carbon electrode modified with poly(pyrrole-lactosyl) film (Γ = 3 × 10^−9^ mol cm^−2^) before (line) and after (dashed line) incubation with PNA (8.3 × 10^−6^ mol L^−1^) in 0.1 mol L^−1^ phosphate buffer (pH = 7.2).** Scan rate at 0.1 V s^−1^.

The possible binding of PNA onto the polymerized lactose and MAA onto the polymerized 3′-siallyllatose was investigated by cyclic voltammetry measurements. Two redox probes exhibiting different molecular size were used: ruthenium hexamine and hydroquinone. PNA and MAA agglutinins were incubated on poly(pyrrole-lactosyl) and poly(pyrrole-3′-sialyllactosyl) electrodes, respectively, for 45 min. This incubation time was determined by SPR (Gondran et al., 2008). Electrodes were then rinsed with phosphate buffer. Figure 4 show the voltammograms of these probes (2 × 10^−3^ mol L^−1^) in phosphate buffer obtained at poly(pyrrole-lactosyl) electrodes before and after PNA incubation. In both cases, a decrease in peak system intensity and an increase in Δ*E*_*p*_ values were observed after the lectin incubation. For instance, the cathodic peak current density for ruthenium hexamine changes from −884 to −835 A cm^−2^ while Δ*E*_*p*_ increases from 80 to 170 mV (Figure 4A). Concerning the hydroquinone couple, the anodic peak current density decreases from 559 to 467 A cm^−2^ and Δ*E*_*p*_ increases from 730 to 1010 mV (Figure 4B). It should be noted that a similar evolution was recorded after the incubation of MAA onto the poly(pyrrole-3′-sialyllactosyl) electrode (data not shown). These phenomena clearly indicate an increase in steric hindrances towards the permeation of these redox probes and hence reflect the specific binding of PNA or MAA on their corresponding polymer. It should be noted that the voltammogram changes due to the anchoring of lectin at the polymer surface are greater with the neutral and smaller probe (hydroquinone) than with ruthenium hexamine. Hydroquinone probe was therefore selected for further study.

### Permeabilities of poly(pyrrole-lactosyl)/PNA and poly(pyrrole-3′-sialyllactosyl)/MMA interfaces

**Figure 5:**
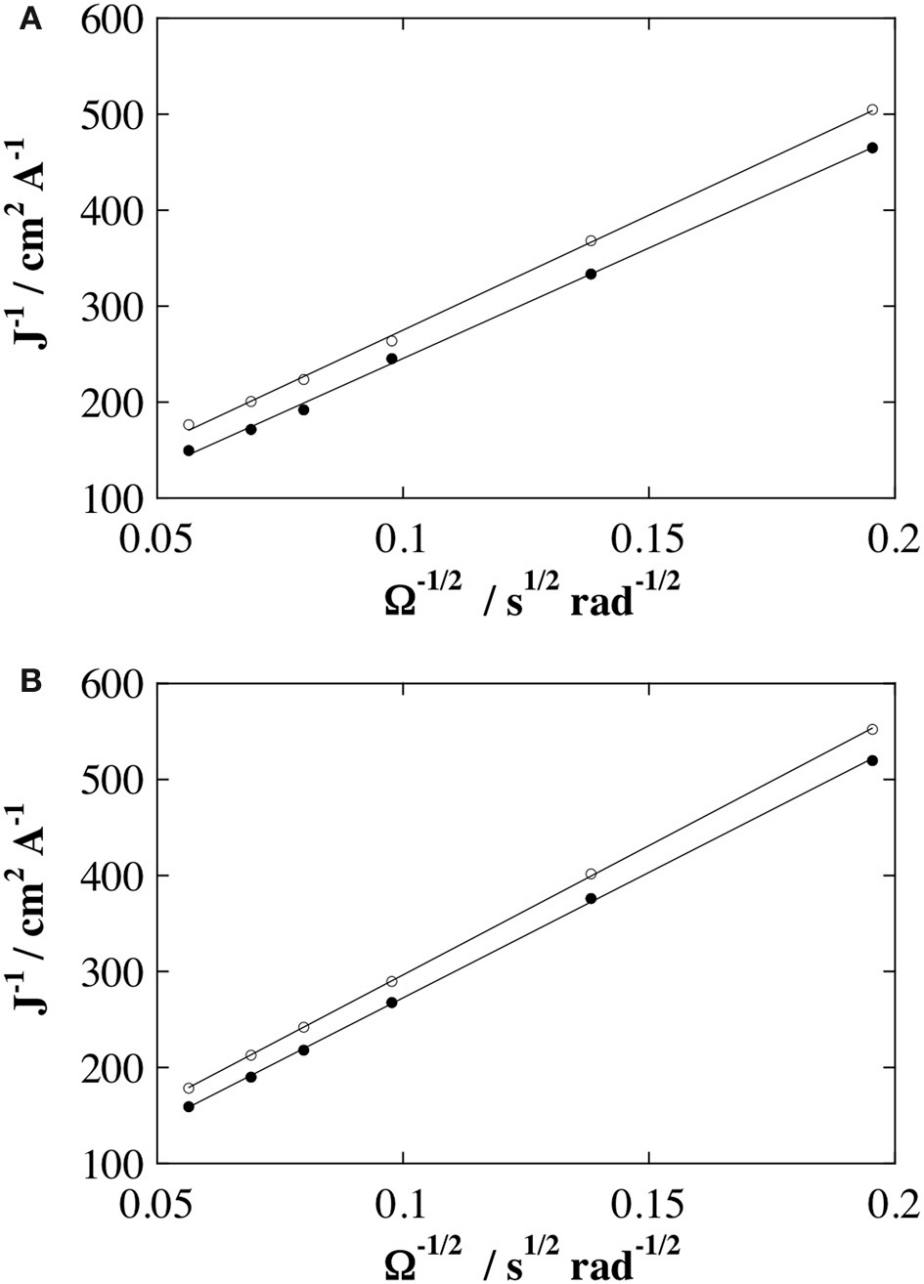
**Koutecky-Levich plots obtained with stationary curves at a scan rate of 5 × 10^−3^ V s^−1^ in the presence of 2 × 10^−3^ mol L^−1^ of hydroquinone in 0.1 mol L^−1^ PB (pH 7.2), the rotating disk voltammograms were recorded from 0.0 to 1.00 V vs. Ag/AgCl at different rotation speeds (from 250 to 3000 rpm) on glassy carbon electrode; before (black circle) and after (empty circle) incubation of 8.3 × 10^−6^ mol L^−1^ of specific lectin (PNA or MAA). (A)** Poly(pyrrole-lactosyl) film (Γ = 2.1 × 10^−9^ mol cm^−2^) (black circle) and poly(pyrrole-lactosyl) film + PNA (empty circle). **(B)** Poly(pyrrole-3′-sialyllactosyl) film (Γ = 2.8 × 10^−9^ mol cm^−2^) (black circle) and poly(pyrrole-3′-sialyllactosyl) film + MAA (thin line).

The evolution of the electrode interface was investigated by linear sweep voltammetry using vitreous carbon rotating disk electrode (RDE) with hydroquinone (2 × 10^−3^ mol L^−1^) as a redox probe in phosphate buffer. Voltammograms were recorded from 0.00 to 1.00 V at 5 × 10^−3^ V s^−1^ in order to involve the potential range of hydroquinone oxidation. Steady-state limiting currents were recorded for the poly(pyrrole-lactosyl) and the poly(pyrrole-3′-sialyllactosyl) electrodes before and after incubating with PNA and MAA, respectively. Figure 5 shows the resulting Koutecky–Levich curves corresponding to reciprocal of the limiting current density vs. reciprocal of the square root of the rotating rate. The permeability of a film, *P*_*m*_, depends on the redox probe and on the membrane according to:

(1) $$P_{m}=\frac{D_{m}K}{\delta }$$

with *D_*m*_* is the diffusion coefficient of the redox probe into the membrane, *K* is the partition equilibrium constant of redox probe between solution and membrane and δ is the thickness of the membrane. The Koutecky–Levich curves were treated by the Equations 2–4 introduced by Gough and Leypold (Gough and Leypoldt, 1979, 1980) that describe the variation of steady state limiting current density *J*_*lim*_ with the mass transport for a rotating disk electrode coated with a membrane:

(2) $$\frac{1}{J_{lim}}=\frac{1}{J_{s}}+\frac{1}{J_{m}}$$

(3) $$J_{s}=0.62n\;F\;C^{{}^{\circ}}\;D_{s}^{2/3}\;\upsilon^{-1/6}\;\Omega^{1/2}$$

(4) $$J_{m}=\frac{n\;F\;D_{m}\;K\;C^{{}^{\circ}}}{\delta }$$

with *n* is the electron number (2 for hydroquinone), F the Faraday constant, *C*° and *D*_*s*_ the concentration and the diffusion coefficient of redox probe in the bulk of the solution, ν the kinematic viscosity of the solution, Ω the rotation rate of the RDE. In the absence of a membrane, the first term in Equation 2 does not exist and the straight line passes throught zero and is therefore characteristic of the diffusion on the substrate in the bulk solution (Levich current, Equation 3). In the presence of a membrane, the positive intercept of the straight lines depend on the permeability of the films according to:

$$\frac{1}{J_{\lim}}\left(\Omega \to \infty \right)=\frac{1}{J_{m}}$$

Table 1 gives the mean values of permeability obtained with three electrodes of each type (poly(pyrrole-lactosyl) or poly(pyrrole-3′-siallyllactosyl)). In accordance with their similar monomeric structures, it appears that the two polymers have similar permeability values, namely 0.18 and 0.22 cm s^−1^ for poly(pyrrole-lactosyl) and poly(pyrrole-3′-sialyllactosyl), respectively. Such range of permeability is higher than those previously reported for other poly(N-substituted-pyrroles) reflecting the high hydrophilic character of these polymers (Ionescu et al., 2004; Giroud et al., 2009; Xu et al., 2012). The latter should promote the polymer swelling in water and hence facilitate the permeation of probes. As expected, after the incubation stage with target protein and a careful rinsing, the permeability decreases demonstrating an efficient recognition process between polymerized saccharides and lectins. A 40% loss of permeability is observed for poly(pyrrole-lactosyl)/PNA interface while the loss is 55% for poly(pyrrole-3′-siallyllactosyl)/MMA interface.

**Table 1:** **Permeability, values of equivalent circuit elements obtained for fitting of the experimental data and impedance modulus at 35 Hz for poly(pyrrole-lactosyl) and at 11.5 Hz for poly(pyrrole-3′sialyllactosyl)**.

	**P_m_/cm s^−1^**	**R_ct_/Ω cm^2^**	**C_dl_/μF cm^−2^**	**f°/Hz**	α	**R_δ_/Ω cm_2_**	**τ/s**	**Z/Ω cm^2^**
**Poly(pyrrole-lactosyl) film**	0.18 ± 0.02	77 ± 3	7.5 ± 0.3	276	0.89	30 ± 1	0.133	90 (to 35 Hz)
**Poly(pyrrole-lactosyl) film + PNA lectin**	0.11 ± 0.02 *(*− *40%)*	97 ± 3 *(*+ *26%)*	7.4 ± 0.3 *(*− *1%)*	222 *(*− *25 %)*	0.89	33 ± 1	0.158	109 (to 35 Hz) *(*+ *21 %)*
**Poly(pyrrole-3′-sialyllactosyl) film**	0.22 ± 0.02	93 ± 3	7.7 ± 0.3	223	0.83	28 ± 1	0.114	110 (to 11.5 Hz)
**Poly(pyrrole-3′-sialyllactosyl) film + MAA lectin**	0.10 ± 0.02 *(*− *55 %)*	131 ± 4 *(*+ *41 %)*	7.6 ± 0.3 *(*− *1 %)*	131 *(*− *41 %)*	0.84	29 ± 1	0.126	143 (to 11.5 Hz) *(*+ *30 %)*

*Relative change between parameters, before and after incubation of the specific lectin, is given in italic*.

### Electrochemical impedimetric characterization of poly(pyrrole-lactosyl)/PNA and poly(pyrrole-3′-sialyllactosyl)/MMA interfaces

**Figure 6:**
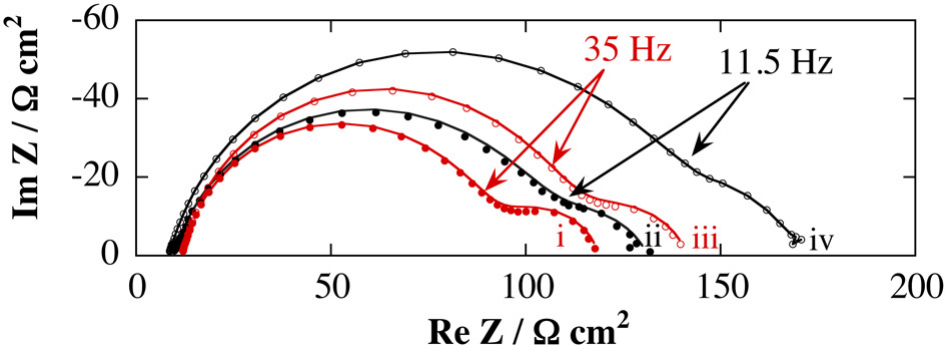
**Nyquist plot of impedance spectra obtained in the presence of 2 × 10^−3^ mol L^−1^ of hydroquinone in 0.1 mol L^−1^ PB (pH 7.2); before and after incubation of 8.3 × 10^−6^ mol L^−1^ of specific lectin (PNA or MAA).** The frequency sweep was from 50 kHz to 0.01 Hz. Impedance measurements were performed at 0.35 V vs. SCE with AC amplitude of 5 mVrms at a rotation rate of 3000 rpm. (i) poly(pyrrole-lactosyl) film (Γ = 2.1 × 10^−9^ mol cm^−2^); (ii) poly(pyrrole-3′-sialyllactosyl) film (Γ = 2.0 × 10^−9^ mol cm^−2^); (iii) poly(pyrrole-lactosyl) film + PNA; (iv) poly(pyrrole-3′-sialyllactosyl) film + MAA. Experimental (dots) and fitted data (lines) are presented.

The possibility of using the poly(pyrrole-lactosyl) or poly(pyrrole-3′-siallyllactosyl) for the impedimetric transduction of the lectin detection, was investigated with hydroquinone (2 × 10^−3^ mol L^−1^) as a redox probe in phosphate buffer. EIS was thus performed at 0.35 V/SCE corresponding to the oxidation of hydroquinone for characterizing the polymer and the lectin binding (Figure 6). After incubating with its specific lectin, each modified electrode presents an increase in impedance reflecting the lectin binding.

**Figure 7:**
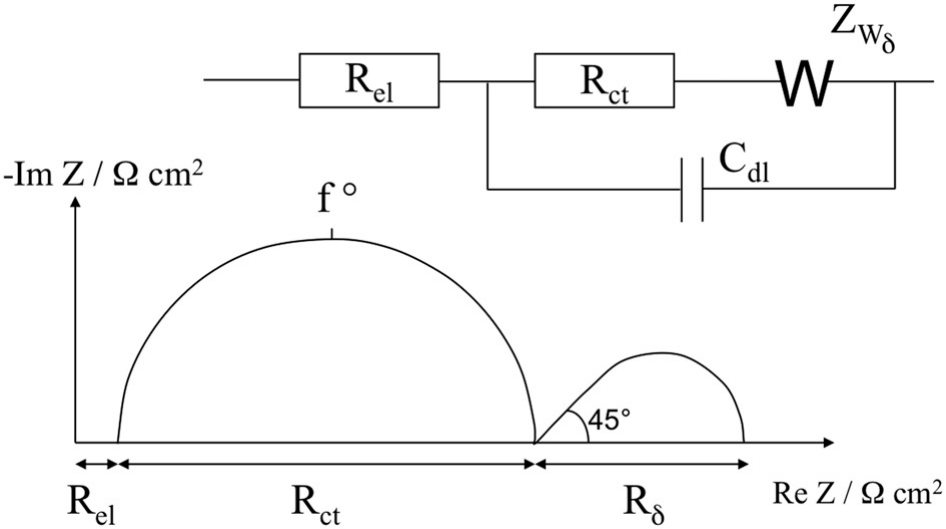
**Equivalent circuit used to fit the experimental data and schematic representation of Nyquist plot corresponding to this equivalent circuit**.

An equivalent circuit was designed to fit the experimental impedance values (Figure 7). The circuit includes the ohmic resistance, *R*_el_, of the electrolyte solution, the electronic charge transfer resistance, *R*_ct_, in series with the finite length Warburg, *W*_δ_, and in parallel with the double layer capacitance, *C*_dl_ (Barsoukov and Macdonald, 2005). The high frequency semicircle of the Nyquist diagram corresponds to the charge transfer resistance in parallel of double layer capacitance. The low frequency loop is attributed to the finite length Warburg due to the diffusion of hydroquinone. We used constant phase element (CPE) instead of capacitance, to account of surface inhomogeneity on the modified electrode (Daniels and Pourmand, 2007). The impedance of a CPE is given by:

$$Z_{\text{CPE}}=Q^{-1}{\left(j\omega \right)}^{-\alpha }$$

where *Q* is the amplitude of the CPE, ω is the angular frequency and α is the exponent which is a real number that varies between 0 and 1. When α = 1, purely capacitive behavior is observed (i.e., *Q* = *C*). The lines passing through the data points in Figure 6 represent the fits using this circuit, its superposition on the data points indicated a good match of the model with the measured data. Table 1 shows values of the equivalent circuit elements obtained by the experimental data fitting.

The comparison of the polymer characteristics before the incubation stage with lectin shows a lower charge transfer resistance (*R*_ct_) for poly(pyrrole-lactosyl) (77 against 93 Ω cm^2^) combined with a higher relaxation frequency (f°) (276 against 223 Hz). Moreover, the α parameter that reflects the homogeneity of the surface of the modified electrode (Barsoukov and Macdonald, 2005), is more important for poly(pyrrole-lactosyl) than for poly(pyrrole-3′-siallyllactosyl) (0.89 against 0.83). All these observations suggest that electron transfer of hydroquinone oxidation is facilitated with the poly(pyrrole-lactosyl) film through better homogeneity and electroactivity (i.e. α higher, *R*_ct_ lower and f° higher). This is corroborated by the higher electroactivity of the poly(pyrrole-lactosyl) film observed previously by cyclic voltammetry (Figure 3A). Other parameters such as the double layer capacitance or the diffusion resistance *R*_δ_ and diffusion relaxation time, τ, are similar for the two polymers.

After incubating with the target lectin, it appears that *R*_ct_ increases from 77 to 97 Ω cm^2^ for PNA and from 93 to 131 Ω cm^2^ for MAA. In addition, the relaxation frequency decreases from 276 to 222 Hz for PNA and from 223 to 131 Hz for MAA. This evolution of both parameters is in good agreement with the slowdown of electron transfer between the modified electrode and hydroquinone due to the steric hindrances generated by the immobilized lectins. The value of the α parameter is almost constant indicating that the lectin binding does not affect the surface roughness. This suggests that the surface is covered with a lectin layer that replicates the roughness of the polymer. Surprisingly, the double layer capacitance remains unchanged in the presence of lectin. While *R*_δ_ and τ values are weakly affected. It seems that the diffusion layer is little influenced by the presence of lectin. A similar behavior was previously reported for the operating principle of an impedimetric DNA sensor with hydroquinone as redox probe (Baur et al., 2010). In summary, the increase in *R*_ct_ remains the most sensitive parameter to detect the molecular recognition between lectin and polymerized saccharides and hence the lectin immobilization. Taking into account that *R*_ct_ increase was 26% for poly(pyrrole-lactosyl)/PNA, and 41% for poly(pyrrole-3′-sialyllactosyl)/MAA, the impedimetric approach seems more sensitive with poly(pyrrole-3′-sialyllactosyl).

The control experiments were carried out in order to investigate the specificity of these two systems. To determine the possible non-specific adsorption of lectin onto the modified electrode, poly(pyrrole-lactosyl) electrodes were incubated with MAA and poly(pyrrole-3′-sialyllactosyl) electrodes were incubated with PNA. After the incubation stage of MAA with poly(pyrrole-lactosyl), the relative variation of *R*_ct_ is +5.6% whereas the *R*_ct_ increase for the detection of PNA was +26%. In the same vein, after the incubation stage of PNA with poly(pyrrole-3′-sialyllactosyl), the relative variation of *R*_ct_ is only +2.5% whereas the specific lectin, MAA induced a *R*_ct_ increase of 41%. Such results unambiguously demonstrate the specificity of the lectin binding onto the polypyrrolic films. It should be noted that addition of MAA onto poly(pyrrole-lactosyl) film leads to higher non-specific interactions than PNA incubated with poly(pyrrole-3′-sialyllactosyl). In fact, although MAA interacts specifically with sialyllactose, this lectin is also sensitive to several lactose derivatives and hence may be adsorbed onto poly(pyrrole-lactosyl). In particular, it has been shown that lactose is an inhibitor of MAA lectin with low affinity (Knibbs et al., 1991).

The use of impedimetric detector requires the recording of impedance spectra over a wide frequency range and the modeling of the data. An attractive alternative may be to develop measurements at a single frequency. It appears that the biggest change of the impedance modulus after the incubation stage of PNA with poly(pyrrole-lactosyl) electrode was detected around 35 Hz for the poly(pyrrole-lactosyl) (109 against 90 Ω cm^2^). This frequency (35 Hz) is at the end of the semicircle (Figure 6). The relative variation of the impedance modulus, Z_35 Hz_, is 20%, while the relative change in the *R*_ct_ was 26%. After incubating with MAA, the poly(pyrrole-3′-sialyllactosyl) electrode provides the biggest change at 11.5 Hz (143 against 110 Ω cm^2^). As expected, this lectin/polymer system leads to a lower detection frequency since this system presents a lower of f° value. The relative variation of the impedance modulus, Z_11.5 Hz_, is then 30%, while the relative change of *R*_ct_ was 41%. Control experiments provide a variation of impedance modulus of 4.3% for poly(pyrrole-lactosyl) incubated with MAA and 1.9% for poly(pyrrole-3′-sialyllactosyl) with incubated PNA. These results corroborate the possibility to develop conductivity detection (at a single frequency) of lectins.

## Conclusion

In this report, we demonstrated the specific anchoring of lectins onto poly(pyrrol-lactosyl) and poly(pyrrole-3′-sialyllactosyl) films by cyclic voltammetry and rotating disk experiments. Beside their reproducibility and homogeneity, the main advantage of these polymers lies in their permeable and hydrophilic character. These characteristics were exploited for the efficient detection of lectin binding by impedance transduction. It is thus expected that such electrogenerated poly(pyrrole-saccharide) films combined with EIS transduction will be useful for the development of impedimetric sensors for the label-free detection of lectins.

### Conflict of interest statement

The authors declare that the research was conducted in the absence of any commercial or financial relationships that could be construed as a potential conflict of interest.
